# Re: Understanding quality of life impact in people with retinal vein occlusion: a qualitative inquiry

**DOI:** 10.1111/cxo.12906

**Published:** 2019-04-24

**Authors:** Mary‐Therese Monaghan, Claire Steenson, Michael A Williams

**Affiliations:** ^1^ Dundee Medical School (now affiliated to NHS Greater Glasgow and Clyde) Dundee UK; ^2^ Northern Ireland Medical and Dental Training Agency Belfast UK; ^3^ School of Medicine, Dentistry and Biomedical Science Queen's University of Belfast Belfast UK

**EDITOR:** The article by Prem Senthil et al.^1^ offers valuable insights into the perspective of patients when they have had a retinal vein occlusion. Two points are worth making that are pertinent to the subject, but not discussed by the authors. First, their results suggest that patients who have had retinal vein occlusion have different health concerns to those with age‐related macular degeneration or diabetic eye disease. In our experience, another difference is that retinal vein occlusion is an unexpected event. Most people with age‐related macular degeneration have heard of the condition, from the press or friends, or at their first attendance, and most patients with diabetes already know that their eyes may be affected. That retinal vein occlusions occur with no warning may therefore lead to a different psychological impact.

Second, the authors cite a participant (interview 17) who said that injections ‘stopped the bleeding’, and this reflects our impression that patients with retinal vein occlusion have a poor understanding of the nature of their condition or its management. We carried out a pilot prospective study (presented at the Irish College of Ophthalmologists Annual Conference 2017) interviewing 20 consecutive retinal vein occlusion clinic patients: three were new and 17 were review patients. The median age was 70.5 years (range 38–88 years). Themes were identified by two authors, and subsequently grouped and refined after discussion.

In response to the question, ‘You have been diagnosed with a retinal vein occlusion. What do you want to know about your eye condition, but don't know or understand at present?’, several themes emerged. First, concern was expressed about the cause of the retinal vein occlusion, specifically in one case that the condition may be cancerous (‘…rogue vessels’), or that it was age‐related macular degeneration (because other patients in the waiting area had this condition). A second theme was on prognosis relating to specific activities: ‘…worried I wouldn't be able to read or drive’, as well as concerns about occupation – a carpenter, for example, asking ‘Can I do this?’. A third theme related to aspects of treatment. Patients wanted to know about alternatives: ‘Should I get glasses?’, and ‘…will an operation help?’. There was a desire to know about treatment effectiveness: ‘Would it have been worse if I didn't get injections?’. Treatment duration was also of interest to patients: ‘…will there be an end to it?’.

Our study lacked the rigour of that of Prem Senthil et al.^1^ However, comments made by the patients revealed a variety of messages that they have picked up on, including specific incorrect misunderstandings and a general uncertainty about treatment. The comments also shone a light on the outcomes that really matter to patients, which are not visual acuity or anatomical parameters.

It is our responsibility as health care professionals to address the patient‐centered perspective highlighted by this work and by Prem Senthil et al.^1^ This is worth doing, as patients with a poor understanding of their retinal vein occlusion may have unjustified anxieties. More work is needed to determine if better understanding leads to improved patient‐related outcomes in retinal vein occlusion.
